# Modeling Mental Health Case-Mix for Quality Improvement—A Comparison of Statistical and AI Models

**DOI:** 10.3390/healthcare13233012

**Published:** 2025-11-21

**Authors:** Jian Gao, Tamara L. Box, Ting Liu, Stacey L. Farmer

**Affiliations:** 1 Office of Productivity, Efficiency and Staffing, Office of Analytics and Performance Integration, Office of Quality and Patient Safety, Department of Veterans Affairs, Washington, DC 20420, USA; 2 Office of Analytics and Performance Integration, Office of Quality and Patient Safety, Department of Veterans Affairs, Washington, DC 20420, USA; 3 Department of Computer Science, Siena University, Loudonville, NY 12211, USA; 4 Albany Stratton VA Medical Center, Department of Veterans Affairs, Albany, NY 12208, USA

**Keywords:** mental health care, outcomes, quality of care, staffing, case-mix, risk adjustment

## Abstract

**Background/Objectives**: With the rising prevalence of mental health (MH) disorders, improving the effectiveness and quality of MH care has become increasingly imperative. To improve patient care outcomes, it is essential to accurately assess staffing needs and compare outcomes across providers to identify best practices. However, without a robust case-mix adjustment system that accounts for disease severity, efforts to measure staffing requirements and evaluate patient outcomes are of limited value. This study aimed to develop such a system by leveraging a large study population, more clinically homogeneous groups, and advanced modeling techniques. **Methods**: In this retrospective population-based study, over two million MH patients (n = 2,088,174) were grouped into 162 clinically homogeneous categories using Clinical Classifications Software Refined (CCSR) to enhance predictive accuracy. We evaluated the performance of four statistical models and four artificial intelligence (AI) models to identify the model that delivered the highest predictive power. **Results**: Among the statistical models, the Box–Cox regression yielded the highest predictive power (R^2^ = 0.42; percent of variation explained [PVE] = 0.300). Among the AI models, CatBoost performed best (R^2^ = 0.458; PVE = 0.311). While the AI models outperformed traditional statistical models, the improvements were modest. Sensitivity analyses confirmed the robustness of these models. **Conclusions**: Both the Box–Cox and CatBoost models demonstrated superior predictive performance compared to those reported in the literature. These findings suggest that a case-mix system based on either model can be used for risk adjustment to optimize staffing levels and benchmark patient outcomes for quality improvement.

## 1. Introduction

Mental health (MH) is a fundamental pillar of overall health, deeply intertwined with physical health and overall well-being. Yet, the prevalence of MH disorders continues to rise [1]. According to the U.S. Substance Abuse and Mental Health Services Administration (SAMHSA) [2], in 2023, 23% of adults in the U.S. experienced a mental disorder, while one in 20 experienced a serious mental illness.

Given the increasing prevalence and complexity of mental health conditions, delivering effective and high-quality care is more important than ever. To improve the effectiveness and quality of care, staffing levels need to be optimized, and patient outcomes should be compared across providers to identify best practices [3,4,5,6]. To assess staffing needs and patient outcomes, a case-mix system that adjusts for disease severity plays an indispensable role.

Although there is extensive literature on case-mix models for overall disease burden and ambulatory care [7,8,9,10], risk-adjustment algorithms specifically tailored to MH remain limited and generally demonstrate low predictive power. For example, in retrospective (concurrent) analyses of actual MH care costs, the highest R-squared reported in the literature was only 0.112 [11,12].

One major challenge in developing a reliable MH case-mix system is the low diagnostic sensitivity and specificity inherent in MH care. Unlike physical conditions such as diabetes or hypertension, which have clear biomarkers like A1C or blood pressure, mental health diagnoses rely on the DSM (Diagnostic and Statistical Manual of Mental Disorders), introducing some degree of diagnostic ambiguity. This lack of clarity contributes to significant variation in treatment options and associated costs, even among patients with the same diagnosis.

Motivated by these challenges and the pressing need to improve patient care, this study aims to develop a robust mental health case-mix system by leveraging: (1) a large study population, (2) actual patient care costs as the outcome, (3) the enhanced specificity of ICD-10-CM codes and the Clinical Classifications Software Refined (CCSR) framework [13], and (4) a range of statistical and AI models.

To enhance the accuracy of the case-mix system, we expanded the CCSR classification into more homogeneous categories and compared the performance of four statistical models and four AI models to evaluate their predictive power. The resulting case-mix system is intended to support health systems in optimizing staffing levels and comparing patient outcomes across providers and hospitals to drive quality improvement.

## 2. Data and Methods

### 2.1. Study Population and Data Source

The study population consists of patients with mental health conditions who received care in fiscal year (FY) 2024 in the Veterans Health Administration (VHA). The VHA is the largest integrated health care system in the U.S., providing care to over 9.1 million Veterans enrolled in the VHA health care system at 1380 health care facilities, including 170 Medical Centers and 1193 outpatient clinics [14]. The VHA health system also maintains a centralized data repository called the Corporate Data Warehouse (CDW), which captures detailed patient demographic, clinical, and cost data.

In this study, we used CCSR developed by AHRQ (Agency for Healthcare Research and Quality) to identify patients with mental health conditions [13]. CCSR classifies patients into 530 clinically homogeneous groups (e.g., hypertension, diabetes, and COPD) based on International Classification of Diseases, 10th Revision, Clinical Modification (ICD-10 CM) codes. Among the 530 groups, 24 of them are for mental health, which include 889 ICD10 codes. After excluding 109 ICD10 codes such as alcohol-related fatty liver disease that are not directly related to mental health care but included in the 24 CCSR categories, we used 780 ICD-10 codes to identify all patients with MH conditions.

Our primary data source was the VHA CDW. We also incorporated clinical and financial data from MH services provided by non-VA providers that were paid by the VHA, as captured by the Integrated Veteran Care Consolidated Data Sets (IVC CDS).

### 2.2. Study Variables and Patient Classification

The outcome or dependent variable was the total mental health care cost at the patient level, serving as a proxy for disease severity or burden. This total cost includes the costs of services provided within the VHA system as well as those provided by non-VHA providers that were reimbursed by the VHA.

The independent variables include age, age groups (for nonlinear effects), sex, and 162 clinical groups, which were derived by expanding the 24 CCSR groups to enhance predictive power. The expansion methodology is described in detail elsewhere [15,16]. Briefly, to increase homogeneity within each clinical group, if a CCSR category included more than 6000 patients (approximately 0.3% of the study population), it was subdivided based on the first digit of the ICD-10 codes. If any resulting subgroup still exceeded 6000 patients, it was further split by the second digit, and so on, until the number of patients would fall below 6000 or the fourth digit was reached. For example, using this process, MBD002 (depressive disorders), which includes 1,544,484 patients, was subdivided into 19 categories.

Although some socioeconomic variables (e.g., private health insurance coverage, marital status, disability ratings) were available, we intentionally excluded them from this study. This is because most existing studies in the literature and commercial case-mix software such as DxCG only use age, sex, and diagnoses as input variables. Using the same input data allows for meaningful comparisons of the predictive performance of different models. Nonetheless, socioeconomic factors can significantly influence health status and should be considered alongside case-mix measures when analyzing staffing levels or comparing patient outcomes.

### 2.3. Statistical Analyses and Artificial Intelligence (AI) Modeling

Multivariate regression is the most widely used analytical tool for developing case-mix or risk adjustment systems [17,18,19]. After fitting the models, the resulting predicted or expected outcome (cost) serves as a metric for disease burden or severity.

Given that the performance of statistical models can vary significantly depending on the data structure, this study evaluated four commonly used models: ordinary least squares (OLS), Gamma regression with a log link, log-linear regression, and Box–Cox regression. Model performance was assessed using R-squared, percent of variation explained (PVE), mean absolute percentage error (MAPE), and mean absolute error (MAE) [19,20,21,22]. These goodness-of-fit metrics were calculated on both the transformed and raw dollar scales. For log-linear and Box–Cox models, the predicted raw dollar costs were obtained using Duan’s nonparametric smearing retransformation to reduce bias [23,24,25].

To guard against overfitting of the models, we randomly split the study population into a development sample (50%) and a validation sample (50%) [26,27]. The models were fitted on the development sample, and the resulting coefficients were applied to the validation sample to generate predicted values. In addition, we conducted extensive sensitivity analyses, including imposing hierarchies on the relevant expanded CCSR categories [11], and testing different development/validation splits (60/40 and 80/20).

In addition to the four statistical models, we evaluated four AI models: Random Forest, LightGBM, XGBoost, and CatBoost. Each AI model has distinct strengths and weaknesses. For example, Random Forest is less prone to overfitting but can struggle with high-cardinality categorical variables and may not capture complex interactions as effectively as boosting methods. LightGBM is highly efficient and fast, especially with large datasets, but it can be sensitive to overfitting. XGBoost delivers strong performance in modeling structured data, but it is slower than LightGBM. CatBoost excels with categorical features and requires less preprocessing, making it particularly user-friendly for health care data, but it may be slower to train.

Although AI has been used in health care to predict future costs or patient outcomes [28,29,30], we are not aware of its application in developing concurrent case-mix models. We evaluated the performance of the four AI models by applying the same metrics used for the statistical models.

All statistical modeling was carried out using SAS Enterprise Guide 8.3, and all the AI models were implemented using Python 3.13.

## 3. Results

The present study included all 2,088,174 patients who had at least one clinical encounter with MH as the principal diagnosis in FY2024 (either with VHA providers or community providers reimbursed by the VHA). As shown in Table 1, the average patient age was 54.1 years, and 18% were female. The average cost per patient was $7135. The cost difference across age groups was substantial (*p* < 0.0001), but the difference was relatively small ($255) between male and female patients, although still statistically significant (*p* < 0.0001).

Table 2 presents the distribution of all MH patients across the 24 CCSR categories. Using these categories and ICD-10-CM codes, we further classified the study population into 162 more clinically homogeneous groups to enhance predictive power. As shown in Table 2, the largest number of patients fell under BMD007 (trauma- and stressor-related disorders), while BMD023 (inhalant-related disorders) had the fewest. Note that patients may have comorbidities across multiple CCSR categories.

In statistical modeling, the development and validation samples (50/50 percent split) produced virtually identical model fit statistics (with R^2^ values differing only at the fourth decimal place), indicating no overfitting. Thus, all model fit statistics reported in this study are based on the validation sample. Given the large sample size (1,044,087 in the development sample), the forward, backward, and stepwise selection procedures yielded virtually identical results. Therefore, only the results based on the stepwise procedure are reported here. For log-linear and Box–Cox models, we also transformed the predicted values back to the raw dollar scale using Duan’s nonparametric smearing estimator and recalculated the model fit statistics.

Although R^2^ is widely used to gauge model fit, it greatly overestimates the percentage of variation explained by the model [20]. Therefore, we also report PVE (Percent of Variation Explained), which is more informative for model selection. As shown in Table 3, OLS yielded an R^2^ of 0.407, however, only 19.3% of the variation in cost was explained by the independent variables.

The Gamma regression with a log link, designed for positively skewed data, was outperformed by three other models. Log-linear regression had reasonable predictive power, but it performed poorly after the predicted values were transformed back to the raw dollar scale. These findings were consistent with other studies [15,16]. Among the four models, Box–Cox regression offered the highest predictive power: R^2^ = 0.458 and PVE = 0.300 on the raw scale.

The model fit statistics of the four AI models are reported in Table 4. As shown, the predictive power of the four models was comparable: Random Forest yielded the lowest predictive power (R^2^ = 0.428, PVE = 0.291), and CatBoost produced the highest (R^2^ = 0.458, PVE = 0.311). Overall, AI models outperformed statistical models in terms of predictive power, but the improvement was small, which is consistent with findings from other studies [28,31,32].

We conducted extensive sensitivity analyses to ensure the robustness of the models. For example, we imposed clinical hierarchies on the expanded CCSR groups, but this had minimal impact on model fit metrics. We also subdivided the 162 expanded CCSR groups to the fifth digit of the ICD-10-CM codes; however, the improvement in predictive power was negligible.

For the Box–Cox regression, we tested multiple values of the transformation parameter (λ) and confirmed that λ = 0.548 yielded the highest predictive performance, as reported in Table 3. Among the AI models, we varied numerical hyperparameters by ±10% to identify the optimal values that maximized predictive power on the validation samples. For instance, in the CatBoost model, after optimization, the final hyperparameters were set as: iterations = 1000, learning_rate = 0.1, depth = 6, loss_function = ‘RMSE’, and verbose = 100.

## 4. Discussion

Mental health disorders are increasingly prevalent worldwide and are responsible for immense suffering, reduced quality of life, adverse physical health, increased mortality, and staggering economic and social costs [33,34,35]. Given the rising MH care needs and limited resources, optimizing MH staffing levels and comparing patient care outcomes across providers to identify best practices have become imperative. However, assessing staffing needs and analyzing outcomes can be counterproductive without a robust case-mix system that accounts for patient disease severity.

By leveraging a large sample size, more granular clinical groups, and best-fit statistical and AI models, this study achieved a predictive power that is four times higher than what has been reported in the literature (R^2^ ≤ 0.112) [11,12]. Our extensive sensitivity analyses demonstrated that the two best performing models (Box–Cox and CatBoost) were robust and generalizable. The significant improvement in predictive power has practical implications for health systems seeking to optimize staffing, allocate resources, and benchmark outcomes in mental health care. By providing a more accurate adjustment for patient complexity, this case-mix system can support fairer comparisons across providers and inform value-based care initiatives.

Notably, although AI models outperformed statistical models in this study, the improvement in predictive power was modest, consistent with findings from previous research [28,31,32]. One possible explanation is that the relationship between the outcome and predictors may not be highly nonlinear or complex, which are conditions under which AI models typically excel. Moreover, the input data (age, sex, and diagnoses) are well-defined and structured, limiting the advantages of AI models that are designed to process complex or unstructured data. Future research could explore hybrid modeling approaches that integrate traditional statistical methods with AI techniques to further improve predictive performance.

Despite the improved predictive power, the study has limitations. The findings are based on data from the VHA, where the majority of patients in the study were male (82%), which could raise concerns about the generalizability of the models to other populations or care settings. However, after excluding sex from the models in our sensitivity analyses, the decrease in R^2^ across different models was less than 0.01. Therefore, the impact on generalizability is likely minimal. In addition, some patients in this study may have used services paid for by Medicare, but the clinical and financial data for those services were not included in this study due to the time lag of data availability. Nonetheless, it is reasonable to assume that the predictive power of the models would increase if Medicare data were available and included.

## 5. Conclusions

In summary, this study presents a scalable, data-driven approach to mental health case-mix modeling that addresses a critical need in the field. By integrating refined diagnostic groupings with advanced statistical and AI techniques, the resulting case-mix system offers a practical and robust method to account for disease severity when assessing staffing requirements and benchmarking patient care outcomes for quality improvement.

## Figures and Tables

**Table 1:** Cost Distribution by Age and Sex.

Age Group (Mean = 54.1)	No. Patients (%)	Mean Cost	Median Cost	Std Dev	Lower Quartile	Upper Quartile
Age < 35	291,334 (14%)	6485	2540	18,630	1127	5584
35 ≤ Age < 45	422,541 (20%)	7680	2538	22,998	1113	5802
45 ≤ Age < 55	339,340 (16%)	7243	2456	21,866	1090	5579
55 ≤ Age < 65	391,784 (19%)	8278	2385	25,605	1018	5632
65 ≤ Age < 75	346,912 (17%)	7807	2165	24,809	935	5137
Age ≥ 75	296,263 (14%)	4571	1858	13,648	862	4007
**Sex**						
Male	1,721,171 (82%)	7179	2235	22,734	983	5115
Female	367,003 (18%)	6924	2776	18,505	1226	6237
**Total**	2,088,174 (100%)	7135	2321	22,050	1019	5319

**Table 2:** Mental Health Patients by CCSR Category (FY2024).

CCSR	CCSR Description	No. Patients
MBD007	Trauma- and stressor-related disorders	1,610,238
MBD002	Depressive disorders	1,544,484
MBD005	Anxiety and fear-related disorders	1,218,420
MBD024	Tobacco-related disorders	580,617
MBD017	Alcohol-related disorders	460,182
MBD026	Mental and substance use disorders in remission	310,070
MBD013	Miscellaneous mental and behavioral disorders/conditions	178,396
MBD014	Neurodevelopmental disorders	170,981
MBD019	Cannabis-related disorders	170,264
MBD003	Bipolar and related disorders	167,225
MBD004	Other specified and unspecified mood disorders	132,989
MBD001	Schizophrenia spectrum and other psychotic disorders	125,877
MBD012	Suicidal ideation/attempt/intentional self-harm	94,005
MBD021	Stimulant-related disorders	88,480
MBD018	Opioid-related disorders	67,392
MBD009	Personality disorders	58,044
MBD025	Other specified substance-related disorders	43,405
MBD006	Obsessive-compulsive and related disorders	29,564
MBD011	Somatic disorders	28,065
MBD008	Disruptive, impulse-control and conduct disorders	16,313
MBD010	Feeding and eating disorders	14,036
MBD020	Sedative-related disorders	10,495
MBD022	Hallucinogen-related disorders	2855
MBD023	Inhalant-related disorders	698

CCSR: Clinical Classifications Software Refined.

**Table 3:** Predictive Power of the Statistical Models.

	OLS	Gamma	Log-Linear		Box–Cox	
	Raw Scale	Raw Scale	Transformed	Raw Scale	Transformed	Raw Scale
R^2^	0.407	NA	0.359	0.001	0.474	0.420
R^2^ Adj	0.407	NA	0.359	0.001	0.474	0.420
PVE	0.193	NA	0.202	0.002	0.249	0.300
MAPE	2.917	6.082	0.112	5.994	0.566	2.853
MAE	6365	7986	0.822	7867	50.77	5528

**Table 4:** Predictive Power of the AI Models.

	Random Forest	LightGBM	XGBoost	CatBoost
R^2^	0.428	0.432	0.441	0.458
PVE	0.291	0.306	0.304	0.311
MAPE	2.465	2.544	2.792	2.495
MAE	5657	5529	5543	5452

## Data Availability

The data presented in this study are available on request from the corresponding author due to the scale and sensitive nature of the data set.
